# Supplementary material for: Factors Associated with Communities’ Satisfaction with Receiving Curative Care Administered by Community Health Workers in the Health Districts of Bousse and Boussouma in Burkina Faso, 2024
Source: Int J Environ Res Public Health. 2024 Aug 30;21(9):1151. doi: 10.3390/ijerph21091151 (PMC11431175; doi:10.3390/ijerph21091151)
Supplement: Supplementary file 1 [file ijerph-21-01151-s001.zip › ijerph-2994324-supplementary.pdf]

Table S1: **Description of the dimensions of quality of care from the consumer's perspective according to BENINGUISSE**

**Dimensions of BENEGUISSE's conceptual model**	**Description of the dimensions**
**Geographical accessibility**	The degree to which the proximity or remoteness of health services suits users in terms of distance/time to get there
**Organizational accessibility**	The extent to which the way services are organised is suitable for users in terms of waiting times, cleanliness and comfort of the premises and the attitude of care staff towards courtesy and respect for human dignity.
**Interpersonal communication**	The carer's ability to understand and explain the patient's health problems and to involve the patient in decisions relating to care
**Technical skills**	The carer's ability to effectively manage the patient's health problems to their satisfaction
**Care continuity**	The way in which distinct elements of patient care are linked together over time and between different providers

Table S2: **Results of bivariate analyses with statistically significant associations**

Level of education * Satisfaction with CBHWs services

	Value	ddl	Asymptotic significance (bilateral)
Pearson chi-square	29.902	5	.000
Likelihood ratio	11.271	5	.046
N valid observations	960		

Accessibility in terms of time (waiting time/scheduling of sessions) * Satisfaction with services offered by CBHWs

	Value	ddl	Asymptotic significance (bilateral)
Pearson chi-square	13.179	4	.010
Likelihood ratio	11.334	4	.023
N valid observations	960		

CBHWs Reception * Confidence in CBHWs prescriptions

	Value	ddl	Asymptotic significance (bilateral)
Pearson chi-square	15.069	3	.002
Likelihood ratio	9.954	3	.019
N valid observations	960		

Cultural accessibility (speaks the same language/same habits, etc.) * Confidence in CBHWs prescriptions

	Value	ddl	Asymptotic significance (bilateral)
Pearson chi-square	38.671	3	.000
Likelihood ratio	16.137	3	.001
N valid observations	960		

Vulnerability status * Confidence in CBHWs requirements

	Value	ddl	Asymptotic significance (bilateral)
Pearson chi-square	35.276	4	.000
Likelihood ratio	13.853	4	.008

N valid observations	960		

* bivariate analysis

Table S3: Logistic regression conditions

Classification table[a,b]

Observed		Preview: Satisfaction with ASBC services: No	Preview: Satisfaction with ASBC services: Yes	Correct percentage
Satisfaction with services provided by CHBWs	No	0	31	.0
	Yes	0	929	100.0
Global percentage				96.8

Equation variables

	B	E.S	Wald	ddl	Sig.	Exp(B)
Constant value	3.400	.183	346.813	1	.000	29.968

Composite tests of model coefficients

	Chi-square	ddl	Sig.
Pas	42.000	18	,001
Block	42.000	18	,001
Model	42.000	18	,001

Classification table[a,b] : Creating correlation matrices and contingency tables
